# Proteomic Characterization of Liver Cancer Cells Treated with Clinical Targeted Drugs for Hepatocellular Carcinoma

**DOI:** 10.3390/biomedicines13010152

**Published:** 2025-01-09

**Authors:** Hezhou Long, Jiafu Zhou, Changxia Zhou, Shuyu Xie, Jingling Wang, Minjia Tan, Junyu Xu

**Affiliations:** 1 School of Pharmaceutical Sciences, Southern Medical University, Guangzhou 510515, China; longhezhou534@zidd.ac.cn (H.L.); zhoujiafu1003@zidd.ac.cn (J.Z.); 2 Zhongshan Institute for Drug Discovery, Shanghai Institute of Materia Medica, Chinese Academy of Sciences, Zhongshan 528400, China; wangjingling317@zidd.ac.cn; 3 School of Chinese Materia Medica, Nanjing University of Chinese Medicine, Nanjing 210023, China; zhouchangxia1023@zidd.ac.cn (C.Z.); xieshuyu1998@163.com (S.X.); 4 State Key Laboratory of Drug Research, Shanghai Institute of Materia Medica, Chinese Academy of Sciences, Shanghai 201203, China

**Keywords:** proteomics, hepatocellular carcinoma, hepatocellular carcinoma-targeted drugs, S/T/Y phosphorylation, drug combination

## Abstract

**Background/Objectives**: Hepatocellular carcinoma (HCC) remains a significant global health concern, primarily due to the limited efficacy of targeted therapies, which are often compromised by drug resistance and adverse side effects. **Methods**: In this study, we utilized a Tandem Mass Tag (TMT)-based quantitative proteomic approach to analyze global protein expression and serine/threonine/tyrosine (S/T/Y) phosphorylation modifications in HepG2 cells following treatment with three clinically relevant hepatocellular carcinoma-targeted agents: apatinib, regorafenib, and lenvatinib. **Results**: Utilizing KEGG pathway enrichment analysis, biological process enrichment analysis, and protein interaction network analysis, we elucidated the common and specific metabolic pathways, biological processes, and protein interaction regulatory networks influenced by three liver cancer therapeutics. The study additionally proposed potential combinational treatment strategies, highlighting a possible synergistic interaction between HCC-targeted drugs and the DNA methyltransferase inhibitor. Furthermore, through the integration of clinical phosphorylation site data, we identified several phosphorylation sites that exhibited higher abundance in tumor tissues compared to adjacent non-tumor tissues. These sites were associated with poor prognosis and elevated functional scores. **Conclusions**: In summary, this study conducted an in-depth analysis of the molecular alterations in proteins and phosphorylation modifications induced by clinical HCC-targeted drugs, predicting drug combination strategies and therapeutic targets.

## 1. Introduction

Liver cancer is the sixth most prevalent malignancy globally and constitutes the fourth leading cause of cancer-related mortality. Hepatocellular carcinoma (HCC) represents the predominant form of liver cancer, comprising over 90% of diagnosed cases. The principal risk factors of HCC include chronic alcohol consumption, non-alcoholic steatohepatitis (NASH) associated with diabetes or obesity, and infection with hepatitis B virus (HBV) or hepatitis C virus (HCV). The global incidence of HCC varies significantly, with projections indicating that by 2040, liver cancer will result in approximately 1.3 million deaths worldwide [1,2]. To date, the United States Food and Drug Administration (FDA) has approved several molecular targeted drugs for targeted therapy in HCC. These agents, including sorafenib, lenvatinib, regorafenib, and cabozantinib, have become the primary systemic therapies for advanced HCC [3,4]. Nonetheless, targeted therapy for liver cancer continues to encounter several challenges, including resistance, limited efficacy, and side effects [5,6]. Many patients developed resistance to sorafenib treatment after six months, and the adverse reactions observed primarily included gastrointestinal, systemic, or dermatological disorders [7]. Lenvatinib is associated with challenges related to resistance and side effects, with some patients reporting diarrhea, decreased appetite, and weight loss and high blood pressure [8]. Regorafenib has shown some efficacy in prolonging survival. However, its use is linked to severe adverse effects, such as hypertension, hand–foot skin reactions, fatigue, and diarrhea [9]. Indeed, a minimal proportion of HCC patients, less than 10%, are cured. Consequently, the majority of patients reach the stage of advanced HCC, at which point systemic therapy remains the sole effective intervention to delay the disease’s natural progression [10].

Proteomics technology, which primarily utilizes liquid chromatography–tandem mass spectrometry (LC-MS/MS), is crucial for the detailed analysis of protein composition and dynamic changes within cells, tissues, and organisms. This encompasses the assessment of protein expression levels, post-translational modifications, and protein–protein interactions. By enabling large-scale evaluations of gene and cellular functions at the protein level, proteomics offers a comprehensive understanding of cellular activities and disease mechanisms, thereby finding increasing applications in cancer research [11,12,13]. Currently, proteomic technologies in liver cancer are primarily applied to biomarker discovery, drug target identification, mechanisms of drug action, resistance and interaction studies, and elucidating the pathogenesis of HCC. The role of proteomics is becoming increasingly important in liver cancer research, contributing to a deeper understanding of disease mechanisms, diagnosis, and treatment. Continuous advancements in proteomic technologies and their flexibility to complement and integrate with other techniques open new and broader avenues for cancer diagnosis and therapy [14,15]. Overall, proteomics has become an important approach in liver cancer research, and its application is driving advancements in the field of liver cancer treatment, particularly when applied to clinical cohort samples. It offers vital insights into the molecular mechanisms underlying liver cancer, facilitates the identification of early diagnostic markers, aids in the recognition of diagnostic and therapeutic targets, enhances prognosis assessment, and supports the development of individualized treatment strategies [16,17,18]. Previous studies have utilized proteomic technology to characterize the proteomic expression profile and phosphoproteomic landscape of early HCC, thereby identifying novel targets for precise liver cancer treatment [19]. Alterations in serine/threonine/tyrosine (S/T/Y) phosphorylation levels in liver cancer encompass multiple critical pathways and biological processes, contributing to the initiation, progression, and malignant transformation of tumors [20,21,22,23]. Activating mutations in β-catenin (CTNNB1) have been identified as a primary driving factor in the pathogenesis of liver cancer. These mutations facilitate the phosphorylation of Akt2 and Cad, thereby promoting the synthesis of pyrimidine nucleotides for DNA replication and contributing to the progression of liver cancer [24]. Proteomics plays a key role in deciphering the mechanisms underlying clinical pharmacotherapy, particularly in targeted therapy and drug resistance investigation [25]. With continuous advancements in technology, the application of proteomics in analyzing clinical drug mechanisms is expected to become increasingly comprehensive and detailed. Apatinib, regorafenib, and lenvatinib are three targeted therapeutic agents that have received considerable attention in recent years for their potential in the treatment of HCC. These pharmacological agents impede critical intracellular signaling pathways within tumor cells, thereby suppressing tumor proliferation, angiogenesis, and metastasis, and are of substantial value in the therapeutic management of HCC [26]. Despite the close relationship between the pathogenesis of liver cancer and protein post-translational modifications, there is still a lack of research concerning the levels of protein expression and phosphorylation modifications following the actions of these three molecular targeted drugs.

In this study, we employed a tandem mass spectrometry-based quantitative proteomics strategy to perform a comprehensive analysis of protein expression profiles in HepG2 cells following treatment with three clinically relevant liver cancer-targeted drugs, identifying a total of 8036 proteins. Differential protein expression was subsequently examined through pathway enrichment and network interaction analyses, which elucidated both the commonalities and distinctions among the three drugs at the pathway level. Furthermore, proteomic prediction methodologies were utilized to identify multiple potential drug combination strategies, providing valuable resources for combination therapy in liver cancer treatment. Building on this foundation, we conducted an in-depth analysis of alterations at the phosphorylation level. By integrating clinical phosphoproteomic data, we identified several phosphorylation sites that were associated with poor prognosis and exhibited higher expression in cancerous tissues compared to adjacent non-cancerous tissues. These sites play a crucial role in tumor progression, proliferation, metastasis, and drug resistance. Overall, our results characterized the molecular alterations in proteins and their post-translational modifications induced by clinically administered liver cancer-targeted drugs and predicted potential combinatory drug strategies along with potential therapeutic targets.

## 2. Materials and Methods

### 2.1. Cell Culture

The HepG2 cell line was cultured in 15 cm dishes and maintained in DMEM (Gibco, Waltham, MA, USA) containing 10% FBS, 100 U/mL penicillin, and 0.1 mg/mL streptomycin. When the cells reached approximately 80% confluence, DMSO (Sigma-Aldrich, St. Louis, MO, USA) was used as the control group, and apatinib, regorafenib, and lenvatinib (Felixbio, Shanghai, China) were used for the experimental groups. According to concentrations reported in published studies [27,28], each experimental group was treated with 2 µM of the drug for 48 h. The cells were incubated at 37 °C with 5% CO_2_.

### 2.2. Protein Extraction and Reductive Alkylation Trypsin Digestion

HepG2 cells were washed twice with cold 1X phosphate-buffered saline (PBS) and lysed with a lysis buffer (8 M urea in 100 mM NH_4_HCO_3_, pH = 8) containing a 1X protease inhibitor, 2X phosphatase inhibitor, 10 mM NaF, 1 mM PMSF, and phosphatase inhibitor cocktails 2 and 3. Following cell scraping, lysates were incubated on ice for 30 min, sonicated, and centrifuged at 21,300 RCF for 10 min at 4 °C. The supernatants were collected, and the protein concentrations were determined using the BCA assay. The protein solutions were reduced with 5 mM dithiothreitol (DTT; Sigma-Aldrich, St. Louis, MO, USA) at 56 °C for 30 min, alkylated with 15 mM iodoacetamide (IAA; Sigma-Aldrich) at 25 °C in the dark for 30 min, and quenched with 30 mM cysteine (Sigma-Aldrich) at 25 °C for 30 min. The protein solutions were then diluted with 100 mM NH_4_HCO_3_ to reduce the urea concentration to below 2 M and digested with trypsin (Hualishi Scientific, Beijing, China) at a 50:1 (protein/enzyme, *w*/*w*) ratio at 37 °C for 16 h. A second digestion with Lys-C (Hualishi Scientific) was conducted at a 100:1 (protein/enzyme, *w*/*w*) ratio at 37 °C for 4 h. The digested peptides were desalted using a Sep-Pak C18 column (Waters, Milford, MA, USA) and dried using a SpeedVac (SPD120, Thermo Fisher Scientific, Waltham, MA, USA).

### 2.3. TMT Labeling

Peptide samples were dissolved in 100 mM triethylammonium bicarbonate (TEAB) buffer, and Tandem Mass Tag (TMT) reagents (Thermo Fisher Scientific, Waltham, MA, USA) were dissolved in 15 μL of acetonitrile (ACN) following the manufacturer’s instructions. The TMT channels were assigned as follows: 126: Control 2; 127N: Control 3; 127C: Control 4; 128N: apatinib1; 128C: apatinib2; 129N: lenvatinib3; 129C: lenvatinib4; 130C: regorafenib2; and 131: regorafenib3. The peptides and TMT reagents were incubated at a 1:2 ratio at 25 °C for 1 h and then quenched with 10% hydroxylamine for 15 min. The labeling efficiency was confirmed to reach 98%, and peptides with different tags were combined, desalted using Sep-Pak C18 columns, and dried under vacuum for subsequent analysis.

### 2.4. HPLC Fractionation

TMT-labeled peptide mixtures were separated on a 1200 Infinity II Series HPLC system (Agilent Technologies, Palo Alto, CA, USA) equipped with a ZORBAX Eclipse XDB-C18 column (Waters, 4.6 × 250 mm, 5 µm particle size). Peptide separation was performed with a 78 min gradient (B%:min: 1:2, 1:4, 3:6, 7:10, 16:25, 21:39, 28:64, 80:72, 98:75, and 100:78) at a flow rate of 1.0 mL/min. The peptides were dissolved in 600 µL of buffer A (2% ACN, 98% H_2_O, pH = 10) and eluted with buffer B (98% ACN, 2% buffer A). Ninety fractions were collected, combined into 20 groups, dried using a SpeedVac, and stored at −80 °C.

### 2.5. LC-MS/MS Analysis

Proteomic analysis was conducted using a Vanquish Neo UHPLC (Thermo Fisher Scientific, Waltham, MA, USA) coupled with an Orbitrap Ascend mass spectrometer (Thermo Fisher Scientific, Waltham, MA, USA) equipped with a DNV75150PN-C18 column (Thermo Fisher Scientific, 75 µm ID × 25 cm length, 2 µm particle size). Dried peptides were dissolved in buffer A (100% H_2_O, 0.1% formic acid) and eluted with buffer B (80% ACN, 20% H_2_O, 0.1% formic acid). The peptides were eluted using a 110 min gradient (B%:min: 5:0, 7:0.5, 30:98, 50:103, 99:106, and 99:110) at a flow rate of 300 nL/min. Data-dependent acquisition was performed using Xcalibur software (version 4.5). The mass spectrometry parameters were set as follows: for MS1 full scans, an Orbitrap detector with a resolution of 120,000 was used, with a scan range of 400 to 1600 *m*/*z*. Automatic gain control (AGC) was set at 8 × 10^5^, and the maximum injection time was set to 50 ms. For MS2, an Orbitrap detector with a resolution of 15,000 was used, with a scan range set from 110 to 2000 *m*/*z*. Precursor ions were fragmented using high-energy collisional dissociation (HCD) mode with a normalized collision energy of 35%. The AGC and maximum injection time were set to 1 × 10^5^ and 27 ms, respectively. Additionally, dynamic exclusion was set to 30 s, and the isolation window was set to 0.7 *m*/*z*.

### 2.6. Database Searching

The raw mass spectrometry data generated by LC-MS/MS were analyzed using MaxQuant software (version 2.4.14.0) against the UniProt human proteome database (downloaded in 19 September 2023, containing 20,411 sequences). Enzyme specificity was set to trypsin/P, allowing up to two missed cleavages. The false discovery rate (FDR) for proteins, peptides, and sites was set at 1%. For proteomics data, the fixed modification was Carbamidomethyl (C), and variable modifications included acetylation (protein N-term) and oxidation (M) [29,30]. MS2 reporter ions were quantified by setting TMT correction factors. Phosphoproteomics data were acquired directly from deep protein expression profile data, with phosphorylation (S, T, and Y) included as a variable modification. Other fixed and variable modifications remained consistent with the proteomic analysis. Phosphorylation sites with a localization probability greater than 0.75 were selected for further bioinformatics analysis.

### 2.7. Differential Protein and Phosphorylation Site Analyses

Differential protein analysis: Proteins identified only by site, as well as reverse or potential contaminant proteins, were removed. To ensure data comparability, the intensity of each channel was normalized to the median of the same channel and then log2-transformed. Student’s *t*-test was used to evaluate the differences in protein expression levels between the control group and drug-stimulated groups, resulting in *p*-values. Then the average channel intensities for the control and each of the three drug-stimulated groups were separately calculated, and fold-change (FC) values comparing the drug-treated groups to the control group were determined. Proteins with *p* < 0.05 and FC > 1.2 were defined as upregulated, while those with *p* < 0.05 and FC < 0.83 were defined as downregulated.

Differential phosphorylation site analysis: Reverse or potential contaminant proteins as well as phosphorylation sites with a localization probability of <0.75 were removed. The intensity of each channel was normalized using protein expression profile data and then log2-transformed, with the remaining data processed in the same manner as for differential protein analysis. Student’s *t*-test was employed to evaluate the differences in phosphorylation modification levels between the control and drug-stimulated groups. Phosphorylation sites with *p* < 0.05 and FC > 1.2 were considered upregulated, while those with *p* < 0.05 and FC < 0.83 were considered downregulated [31,32].

### 2.8. Enrichment and Protein Interaction Network Analyses

KEGG pathway and biological process (BP) enrichment analysis: Gene names of the upregulated and downregulated differentially expressed proteins, as well as proteins with significantly altered phosphorylation sites, were separately input into the WebGestalt website. The Over-Representation Analysis (ORA) method was selected, with Homo sapiens as the organism. The relevant functional database was used to investigate KEGG pathways and biological processes. A significance threshold of FDR < 0.05 was applied.

Protein interaction network analysis and annotation: The gene names of upregulated and downregulated differentially expressed proteins and proteins with significantly altered phosphorylation sites were entered into the STRING database (version 12.0), selecting Homo sapiens as the organism, to perform protein–protein interaction network analysis. The resulting data were imported into Cytoscape software (version 3.10.2). The Molecular Complex Detection (MCODE) plugin in Cytoscape was then used to analyze highly interconnected regions. Gene names associated with clusters were submitted to STRING for KEGG or Gene Ontology (GO) annotation.

### 2.9. Potential Drug Combination Prediction

Proteomics data from our study were integrated with proteomics data from 56 drugs in the ProTargetMiner database (https://protargetminer.genexplain.com/, accessed on 30 September 2024) [33] and 875 drugs from Supplementary Table S1 in Mitchell, D.C et al. (2023) [34] to identify overlapping genes. Spearman correlation analyses for these overlapping genes were conducted using the cor.test() function from the base stats package in R software (version 4.4.0), yielding correlation coefficients and *p*-values.

### 2.10. Analysis of Potential Phosphorylation Functional Sites

Integrative analysis of clinical phosphorylation site data: Using the HBV-related clinical phosphorylation site data from the supplementary materials of Gao Qiang, Zhu Hua, Dong Ling, et al. (2019) [35], the Wilcoxon signed-rank test was performed in R software (version 4.4.0) to identify clinical phosphorylation sites with increased expression in tumor tissues compared to adjacent non-tumor tissues (T/N > 1.5, *p* < 0.05). Additionally, we divided data from HBV patients into two groups, using the median as a cutoff to define high and low protein expression levels. A log-rank test was then conducted using the survdiff() function from the survival package (version 3.7.0) in R software (version 4.4.0) to identify phosphorylation sites associated with poor prognosis (*p* < 0.05). Our data on upregulated phosphorylation sites were overlapped with the identified clinical phosphorylation sites to discover potential functional phosphorylation sites for three HCC-targeted drugs.

Phosphorylation site functional scoring: After identifying potential phosphorylation sites for the three HCC-targeted drugs, we obtained functional scores for the human phosphoproteome from Supplementary Table S3 of Ochoa et al. (2019) [36]. These scores were then applied to our phosphorylation sites, and the results were visualized using GraphPad Prism software (version 9.5.1).

### 2.11. Analysis of Protein Structural Models

Homologous protein structure models for thymidine kinase 1 (TK1) and ribonucleotide reductase subunit M2 (RRM2) were constructed using their amino acid sequences. The TK1 model was obtained from AF-P04183-F1 and the RRM2 model from AF-P31350-F1-v4, both identifiers from the AlphaFold database [AlphaFold DB]. Visualization was conducted using the Open-Source PyMOL software (Version 3.1.0a0, Schrödinger, LLC, New York, NY, USA).

### 2.12. Western Blot Analysis

The cell lysates were combined with loading buffer, boiled at 99 °C for 5 min, and placed on ice for 5 min. This cycle was repeated three times. Protein samples were separated using sodium dodecyl sulfate-polyacrylamide gel electrophoresis (SDS-PAGE) and transferred to a nitrocellulose (NC) membrane at 100 V for 90 min. The membrane was blocked with PBST containing 5% BSA at room temperature for 1 h, followed by incubation with a pan-phosphorylation antibody (1:1000, catalog number AP1067, ABclonal, Wuhan, China) at 4 °C overnight. The membrane was washed three times with PBST, then incubated with a secondary antibody (1:5000) at room temperature for 1 h. Finally, the membrane was washed five times with PBST, and detection was performed using chemiluminescence (Chemiscope 6200, Clinx, Shanghai, China).

### 2.13. Statistical Analysis

Based on the conventional statistical analysis methods in previous proteomics articles, Student’s *t*-test was used to statistically evaluate differentially expressed proteins and phosphorylation sites [37,38,39]. The criteria for defining upregulated proteins or phosphorylation sites were *p* < 0.05 and FC > 1.2, whereas *p* < 0.05 and FC < 0.83 were used to define downregulated proteins or phosphorylation sites. The Wilcoxon signed-rank test was used to evaluate the differential expression of phosphorylation sites in tumor tissues and adjacent non-tumor tissues [40], with *p* < 0.05 and T/N > 1.5 defining upregulated sites with higher expression in tumor tissues compared to adjacent non-tumor tissues.

### 2.14. Software and Visualization

All data were analyzed and visualized using R software (version 4.4.0), GraphPad Prism (version 9.5.1), and Microsoft Office. Based on the quantitative analysis results, the proteins with the highest and lowest abundances were identified by the median of protein regulation across three HCC-targeted drugs, and these data were visualized using R software. KEGG pathway and biological process (BP) enrichment analyses were conducted using the WebGestalt website (https://www.webgestalt.org/, accessed on 5 September 2024) [41], and the results were plotted using the ggplot2 package in R software. Protein–protein interactions were analyzed using the STRING database (version 12.0) [42]. The resulting data were then imported into Cytoscape software (version3.10.2) [43] for further analysis and visualization, with MCODE [44] being employed to analyze highly connected clusters.

## 3. Results

### 3.1. Drug-Perturbated Proteomics in Liver Cancer HepG2 Cell Line

**Figure 1:**
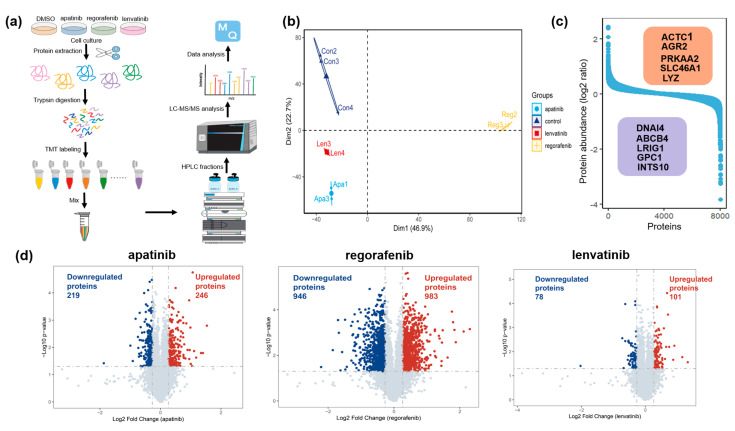
Drug-perturbated proteomics in liver cancer HepG2 cell line. (**a**) Workflow for TMT-based proteomic analysis with apatinib, regorafenib, and lenvatinib treatments in the HepG2 cells. (**b**) Results from the principal component analysis of proteomic data. (**c**) Overview of protein expression after treatment with three liver cancer-targeted drugs, showing the dynamics of protein abundance (log2 ratio). (**d**) Volcanic map of differentially expressed proteins in HepG2 cells treated with apatinib, regorafenib, and lenvatinib.

To elucidate the molecular dynamics of HCC cell lines in response to first- and second-line clinical therapies, we employed a TMT-based quantitative proteomics strategy. Following treatment of the HepG2 cells with three targeted agents for 48 h, cell lysates were obtained using a lysis buffer. Proteins were extracted and subsequently digested into peptides using trypsin and Lys-C. The peptides were then labeled with TMT reagents and separated via an HPLC system. The dried peptides were desalted using C18-coated solid phase to obtain purified peptides, which were finally analyzed using LC-MS/MS to generate comprehensive proteomic profiles (Figure 1a). Subsequently, a principal component analysis (PCA) was conducted utilizing the results generated by MaxQuant software. The PCA result indicated that all three treatment groups displayed varying extents of biological differences compared to the control group, with the regorafenib-treated group exhibiting notably significant biological differences (Figure 1b). A total of 8036 proteins were quantified across the three drug-treated cell lines, identifying proteins with the highest and lowest abundances based on the median of protein regulation across three different HCC-targeted agents (Figure 1c). A subsequent analysis of protein perturbations, relative to the control group, revealed the identification of upregulated proteins (*p* < 0.05 and FC > 1.2) and downregulated proteins (*p* < 0.05 and FC < 0.83). In the apatinib-treated group, 246 proteins were significantly upregulated, and 219 proteins were significantly downregulated, collectively constituting approximately 5% of the total quantified proteins. The regorafenib-treated group identified 983 upregulated proteins and 946 downregulated ones, representing about 24% of the total quantified proteome. In the lenvatinib-treated group, 101 proteins were upregulated, and 78 proteins were downregulated, accounting for roughly 2% of the total proteins quantified (Figure 1d, Table S1).

### 3.2. Functional Enrichment Analysis of Changed Proteins Treated with Different Drugs

**Figure 2:**
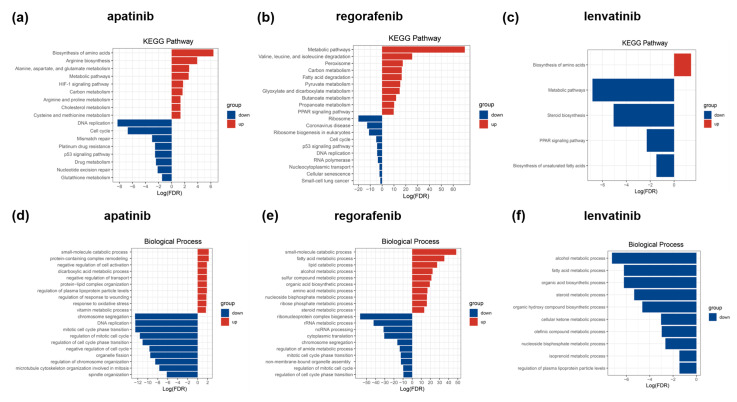
Functional enrichment analysis of changed proteins treated with different drugs. KEGG pathway enrichment analysis of upregulated and downregulated proteins in (**a**) apatinib-treated, (**b**) regorafenib-treated, and (**c**) lenvatinib-treated groups. Biological process analysis of differentially expressed proteins in (**d**) apatinib-treated, (**e**) regorafenib-treated, and (**f**) lenvatinib-treated groups.

Subsequently, we conducted KEGG pathway enrichment analysis on the differentially expressed proteins utilizing WebGestalt. We did not observe consistent enrichment across the three HCC-targeted drugs, prompting a comparison between two pairs of treatment groups. The results indicated that metabolic pathways and carbon metabolism were consistently upregulated in both the apatinib- and regorafenib-treated groups. Similarly, the amino acid biosynthesis pathway was commonly upregulated in both the apatinib- and lenvatinib-treated groups. Conversely, pathways such as DNA replication, the P53 signaling pathway, and the cell cycle were consistently downregulated. Notably, metabolic pathways were included in the downregulated pathway set for the lenvatinib-treated group. The presence of these commonly enriched pathways indicates potential similarities in the mechanism of action among three liver cancer-targeted drugs at the cellular level, highlighting their roles in liver cancer-related therapeutic processes (Figure 2a–c). Additionally, our KEGG pathway analysis results align with previous findings regarding differential protein-enriched pathways in Huh-7 cells treated with the same drugs. In the regorafenib-treated group, upregulated protein-enriched KEGG pathways included metabolic pathways, carbon metabolism, pyruvate metabolism, fatty acid degradation, and valine, leucine, and isoleucine degradation. Downregulated protein-enriched KEGG pathways included DNA replication, the cell cycle, and the P53 signaling pathway. The lenvatinib-treated group exhibited downregulation in protein enrichment associated with metabolic pathways. These results are consistent with previous studies [45].

In the analysis of biological processes, the comparison between the two groups indicated that the upregulated differential proteins in both the apatinib- and regorafenib-treated groups were primarily linked to the metabolic processes involved in small molecule catabolism. In the downregulated protein function enrichment analysis of apatinib- and regorafenib- treated groups, processes such as chromosome segregation, mitotic cell cycle phase transition, the regulation of the mitotic cell cycle, and the regulation of cell cycle phase transition were commonly co-identified (Figure 2d,e). For the lenvatinib-treated group, the analysis revealed no enrichment in the upregulated protein. However, the enrichment analysis of the downregulated proteins was associated with processes involved in alcohol metabolism, fatty acid metabolism, and organic acid biosynthesis processes (Figure 2f). Furthermore, previous proteomic studies on apatinib reported that GO enrichment analysis showed differentially expressed proteins involved in the cell cycle and mitosis. These findings align with our biological process analysis results for the apatinib-treated group [46].

### 3.3. The Protein–Protein Interaction Network Under the Treatment of Different Drugs

**Figure 3:**
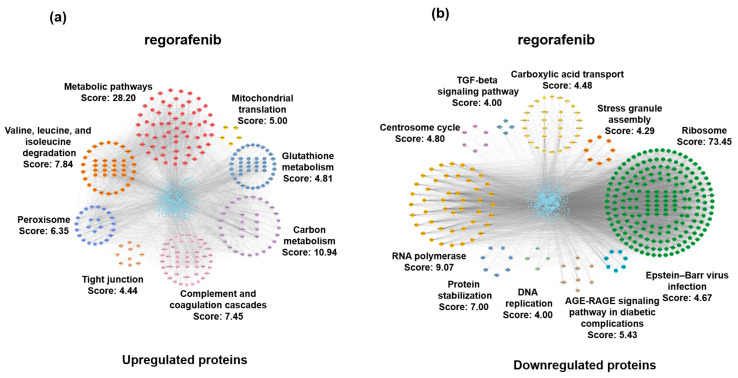
The protein–protein interaction network under the treatment of regorafenib. The protein–protein interaction network of upregulated proteins (**a**) and downregulated proteins (**b**) in the regorafenib-treated group.

To elucidate the potential roles of protein alterations induced by different drug treatments, we used the STRING database to analyze the protein–protein interaction networks of differentially expressed proteins. Subsequently, we utilized the MCODE plugin in Cytoscape to identify clusters of highly interconnected interaction networks. Our results indicated that within the regorafenib-treated group, the upregulated protein interaction network comprised eight distinct clusters. Among these, the clusters with the highest scores were associated with metabolic pathways (score: 28.20), carbon metabolism (score: 10.94), and the degradation of valine, leucine, and isoleucine (score: 7.84) (Figure 3a). Conversely, the downregulated protein interaction network consisted of ten clusters, with the highest scoring clusters pertaining to ribosomes (score: 73.45), RNA polymerase (score: 9.07), and protein stabilization (score: 7.00) (Figure 3b). In the apatinib-treated group, the analysis of the upregulated protein interaction network identified four clusters, with high-scoring clusters related to cholesterol metabolism (score: 8.00) and gap junctions (score: 4.44). For the downregulated protein interaction network in the apatinib-treated group, the high-scoring cluster was related to the cell cycle (score: 41.33) (Figure S1a). Within the lenvatinib-treated group, only one cluster associated with one carbon pool through folate (score: 4.00) was identified within the upregulated protein interaction network. The downregulated protein interaction network revealed clusters predominantly associated with steroid biosynthesis (score: 9.64) (Figure S1b). In summary, these results highlight the regulatory effects of three liver cancer-targeted drugs on various biological pathways and functions at the molecular level.

### 3.4. Proteomic Analysis Predicting Potential Drug Combination Strategy

**Figure 4:**
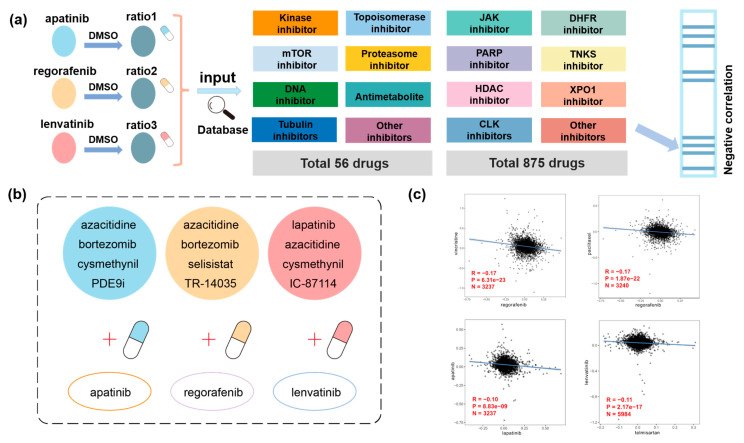
Proteomic analysis predicting potential drug combination strategy. (**a**) Workflow of combination therapy prediction using published proteome profiling data. (**b**) Overview of potential drug combination of three liver cancer-targeted drugs identified through published proteomics data. (**c**) Scatter plots illustrating previously reported drug combinations of three liver cancer-targeted drugs.

Subsequently, we assessed the potential application of our proteomics data for drug combination strategies by utilizing previously reported proteomics data collected from liver cancer cell line A549 [33] and colorectal cancer cell line HCT116 [34], which were treated with 56 or 875 compounds, respectively. We employed a proteomics-based drug combination prediction approach, using the upregulated and downregulated proteomic features under stimulation as input data. Spearman correlation analyses were subsequently conducted to compare our findings with these previously reported proteomic data. Drugs or compounds exhibiting a negative Spearman correlation coefficient (*p* < 0.05, rho < −0.1) were considered as potential candidates for combination therapy (Figure 4a). Our results indicated several drug combination strategies, such as regorafenib, apatinib, and lenvatinib, each showing promising synergistic effects when combined with the DNA methyltransferase inhibitor azacitidine (rho = −0.43, −0.20, −0.12). Additionally, regorafenib and apatinib may exhibit synergistic interactions with the proteasome inhibitor bortezomib (rho = −0.29, −0.13). Similarly, lenvatinib and apatinib, in conjunction with the Isoprenylcysteine carboxyl methyltransferase (ICMT) inhibitor cysmethynil (rho = −0.19, −0.23) indicated potential for combined therapeutic efficacy (Figure 4b).

Previous studies have indicated that the combination of regorafenib with standard-dose vincristine (rho = −0.17) and irinotecan demonstrates clinical efficacy in pediatric patients diagnosed with rhabdomyosarcoma and Ewing’s sarcoma [47]. The combination treatment of regorafenib and paclitaxel (rho = −0.17) was also found to be tolerable and showed substantial efficacy as a first-line therapeutic approach for refractory advanced esophagogastric cancer (EGC) [48]. Additionally, the combination of apatinib and lapatinib (rho = −0.10) or a STING agonist has the potential to significantly enhance sensitivity to apatinib in head and neck squamous cell carcinoma [49]. The concurrent administration of lenvatinib with telmisartan (rho = −0.10) resulted in reduced systemic exposure to telmisartan, indicating potential pharmacokinetic interactions between these two drugs [50]. These reported findings substantiate the reliability of our data and warrant further investigation into the potential drug combinations we have identified (Figure 4c).

### 3.5. Characterization of the Dynamic Phosphorylation Level After Treatment with the Three Drugs in the HepG2 Cell Line

**Figure 5:**
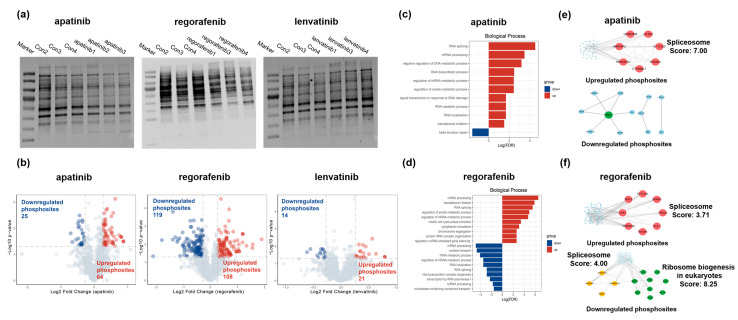
Characterization of the dynamic phosphorylation level after treatment with the three drugs in the HepG2 Cell Line. (**a**) The Western blot analysis of global phosphorylation in HepG2 cells treated with apatinib, regorafenib, and lenvatinib. (**b**) Volcano plots of differential phosphorylation sites based on protein expression profile data for three liver cancer-targeted drugs. Biological process analysis of proteins with significantly altered phosphorylation sites in (**c**) apatinib- and (**d**) regorafenib-treated groups. Protein–protein interaction network of proteins with significantly altered phosphorylation sites in (**e**) apatinib- and (**f**) regorafenib-treated groups.

We proceeded with our investigation into the alterations in phosphorylation modification sites induced by three HCC-targeted drugs. Initially, we utilized Western blot analysis to evaluate the global changes in phosphorylation levels in the HepG2 cell line after drug treatment. Our analysis revealed slight alterations in the phosphorylation levels of whole cell lysates via Western blot analysis following treatment with three liver cancer-targeted drugs (Figure 5a). Subsequently, we explored the potential phosphorylation event using our proteomic data, identifying 1239 phosphorylation sites with a probability exceeding 0.75. By normalizing the phosphoproteomic data by their protein expression level, we identified upregulated and downregulated phosphorylation sites (*p* < 0.05 and FC > 1.2; *p* < 0.05 and FC < 0.83). In the apatinib-treated group, we observed 25 phosphorylation sites exhibiting upregulation and 64 sites showing downregulation at the phosphorylation level. The regorafenib-treated group identified 108 upregulated and 119 downregulated phosphorylation sites. Meanwhile, the lenvatinib-treated group showed 21 upregulated and 14 downregulated phosphorylation sites (Figure 5b, Table S2). Biological process analysis indicated that in the apatinib- and regorafenib- treated groups, proteins with upregulated phosphorylation levels both are associated with RNA splicing, mRNA processing, the regulation of mRNA metabolic process, translational initiation, and the regulation of amide metabolic process. Notably, proteins with downregulated phosphorylation sites in the regorafenib-treated group are also related to RNA splicing, mRNA processing, and the regulation of mRNA metabolic process. Meanwhile, proteins with downregulated phosphorylation sites in the apatinib-treated group were associated with base excision repair (Figure 5c,d).

To investigate the potential roles of phosphorylation modification, we performed a protein–protein interaction network analysis on proteins exhibiting differential phosphorylation. Our results revealed significant enrichment of clusters associated with spliceosomes in both the upregulated phosphorylated proteins from the apatinib-treated group and the up/downregulated proteins from the regorafenib-treated group. This finding aligns with previous research indicating that spliceosome and splice factor alterations, as well as the aberrant expression of oncogenic splice variants, are closely linked to the pathogenesis of hepatocellular carcinoma [51]. Furthermore, within the regorafenib-treated group, the highest score in the downregulated phosphorylation protein interaction network was associated with ribosome biogenesis in eukaryotes (Figure 5e,f). Conversely, no clusters with high scores were enriched in the downregulated phosphorylation protein interaction networks of the apatinib-treated group or in either the upregulated or downregulated networks of the lenvatinib-treated group. In the apatinib-treated group, the network characterized by downregulated phosphorylation proteins identified NCL as the gene with the highest degree of connectivity (Figure 5e). In summary, our investigation into alterations in phosphorylation-modified proteins has elucidated biological processes and protein interactions, facilitating the identification of prospective therapeutic targets and key regulatory sites.

### 3.6. Functional Exploration of the Identified Phosphorylated Substrates

**Figure 6:**
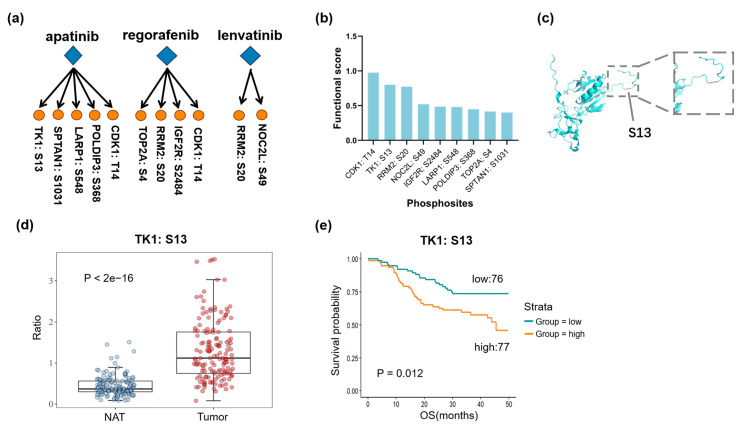
Functional exploration of the identified phosphorylated substrates. (**a**) Overview of functional phosphorylation sites for three liver cancer-targeted drugs based on integrated clinical phosphorylation data. (**b**) Overview of functional phosphorylation sites based on phosphorylation site functionality scoring. (**c**) Visualization of phosphorylation site in the TK1: S13 protein structure model. (**d**) Box plot illustrating differential expression of TK1: S13 in tumors versus NATs. (**e**) Survival probability analysis of phosphorylation site TK1: S13 associated with poor prognosis.

Subsequently, we integrated our phosphorylation site data with previously published clinical phosphoproteomics data [35] related to HBV-associated hepatocellular carcinoma, encompassing 159 patients with paired tumor and adjacent liver tissue samples. This analysis identified several phosphorylation sites that were higher in the tumor tissues compared to adjacent non-tumor tissue and were positively correlated with poor prognosis. Our results showed that five significant sites were identified in the apatinib-treated group: TK1: S13, SPTAN1: S1031, LARP1: S548, POLDIP3: S368, and CDK1: T14; four sites were identified in the regorafenib-treated group: TOP2A: S4, RRM2: S20, IGF2R: S2484, and CDK1: T14; and two sites were identified in the lenvatinib-treated group: RRM2: S20 and NOC2L: S49 (Figure 6a). We also assessed the functional scores of these sites reported in a previous study [36]. Among these, CDK1: T14 exhibited the highest functional score (score: 0.97), followed by TK1: S13, which had the second-highest score (score: 0.80), and RRM2: S20 ranks third (score: 0.77) (Figure 6b). Previous reports have indicated that TK1 contributes to HCC tumorigenesis through its enzymatic activity and the subsequent synthesis of thymidine monophosphate, thereby regrading it as a key driver in HCC development. Similarly, RRM2 can play an important role in the fight against iron death in HCC cells by maintaining the synthesis of intracellular glutathione (GSH) and can be an important marker and target for HCC diagnosis and treatment [52,53]. We focused on the phosphorylation sites TK1: S13 and RRM2: S20 and visualized the protein’s three-dimensional structure based on the amino acid sequence (Figure 6c and Figure S2a). The clinical proteomic analysis revealed that both TK1: S13 and RRM2: S20 were significantly overexpressed in tumors in comparison to normal adjacent tissues (NAT) (Figure 6d and Figure S2b), with higher phosphorylation levels closely associated with poor prognosis in HBV-associated HCC (Figure 6e and Figure S2c). This result indicated that the phosphorylation sites TK1: S13 and RRM2: S20 are pivotal in the progression of liver cancer and may serve as a significant target for research and therapeutic interventions. In conclusion, the analysis of clinical phosphoproteomics data enables the identification of phosphorylation sites that exhibit significant alterations during the progression of liver disease, indicating their potential roles serving as drug targets.

## 4. Discussion

Mass spectrometry-based proteomic strategies have played a crucial role in elucidating drug mechanisms of action and identifying drug targets. These strategies include drug target identification, mechanism of action analysis, target discovery and validation, as well as the assessment of drug safety and efficacy [54]. For instance, plasma proteomics has facilitated predictions regarding therapeutic outcomes for HCC with extrahepatic metastasis and has identified potential therapeutic targets [55]. In conclusion, mass spectrometry-based proteomics has evolved from a technology-driven discipline into an essential analytical tool within life sciences [56]. It has shown considerable potential and value in liver cancer research, offering novel approaches and methodologies for early diagnosis, biomarker discovery, and the identification of therapeutic targets [57,58,59]. The era of precision medicine driven by proteomics has begun with the future poised for even greater breakthroughs.

Our research employed TMT-labeled quantitative proteomics to analyzing the investigate alterations in protein expression and phosphorylation modifications in HepG2 cell lines following treatment with three clinical liver cancer-targeted drugs. By utilizing KEGG pathway enrichment, biological process enrichment analysis, and protein interaction network analysis, we elucidated the pathway processes and biological functions associated with differentially expressed proteins and phosphorylation-modified proteins. Additionally, we incorporated protein expression data from previously published studies, including additional liver cancer cell lines, to enhance the identification of potential drug combinations that may exhibit synergistic effects with three HCC-targeted drugs used in our study. Furthermore, we integrated phosphorylation site data with clinical phosphorylation sites to identify the phosphorylation sites that exhibited higher phosphorylation level in cancerous tissues compared to adjacent non-tumor tissues. These phosphorylated sites were correlated with poor prognosis and possessed high functional scores, indicating their potential as targets for drug therapy. Using high-resolution mass spectrometry-based proteomics, we conducted an in-depth analysis of dynamic alternation in protein expression and phosphorylation states following drug treatment, elucidating their association with the drugs’ mechanisms of action and therapeutic efficacy.

Protein post-translational modifications (PTMs), typically catalyzed by enzymes, serve as critical regulators of protein functionality and are implicated in nearly all cellular processes. PTMs influence protein function by modulating their physical and chemical properties, folding, conformation, stability, and activity. The dysregulation of PTMs is linked to the pathogenesis of various diseases [60,61]. Phosphorylation, as a prevalent PTM, is closely linked to the pathogenesis and targeted therapy of liver cancer. Research has demonstrated that alterations in phosphorylation in HCC affect various biological processes, including cell proliferation, DNA repair, the immune system, and signal transduction. In CTNNB1-mutant HCC, the loss of epithelial phenotype may be attributed to changes in the phosphorylation of proteins associated with actin filament organization, affecting cell polarity and migration. In contrast, TP53-mutant HCC is associated with phosphorylation alterations in proteins related to lipid metabolism and cell cycle control [62]. Studies indicate that immunity-related GTPase M (IRGM) enhances the expression of programmed death-ligand 1 (PD-L1) in tumors by promoting S6K1-mediated phosphorylation of YBX1, representing a novel target for PD-L1 regulation. The PD-L1 contributes to the malignant progression of HCC and immune suppression of CD8+ cytotoxic T lymphocytes (CTLs). Therefore, combining Irgm1 inhibitors with immune checkpoint inhibitors (ICIs) offers a new strategy for HCC treatment [63]. Furthermore, the noncanonical IκB kinase subunit epsilon (IKBKE) promotes hepatocarcinogenesis through the phosphorylation and inactivation of forkhead box A1 (FOXA1), identifying FOXA1 as the true substrate and negative nuclear effector of IKBKE in HCC. This discovery provides a promising strategy for targeting IKBKE in HCC therapy [64]. Phosphorylation mediated by RNA binding motif protein 45 (RBM45) enhances the stability of ASCT2, facilitating the progression of HCC. The RBM45-ASCT2 axis is recognized as an independent prognostic factor in HCC development and serves as a potential drug target [65]. In summary, phosphorylation modifications play a crucial role in the development, progression, and targeted treatment of HCC. Moreover, our phosphoproteomic data identified the phosphorylation site IGF2R: S2484 as being associated with HCC tumorigenesis. Alcohol- and HBV-related HCC (AB-HCC) is described as a unique metabolic subtype characterized by oncogenic cholesterol. Cholesterol directly binds to casein kinase 2 alpha 1 (CSNK2A1), enhancing its activity and leading to the phosphorylation of the insulin-like growth factor 2 receptor (IGF2R) at Ser2484. This cascade reactivates lipid-driven mitochondrial oxidative phosphorylation, generates reactive oxygen species, and perpetuates the positive feedback loop of cholesterol biosynthesis, ultimately resulting in tumorigenesis [66].

Notably, Tan et al. pioneered the elucidation of the regulatory mechanisms associated with lysine 3-hydroxybutyrylation modifications during the process of liver cancer [67]. Yang et al. identified that protein lysine lactylation plays a crucial role in regulating cellular metabolism and may be implicated in the progression of HCC [68]. Additionally, Ma et al. elucidated the critical role of succinyl-CoA: 3-ketoacid CoA transferase 1 (OXCT1) as a lysine succinyltransferase in the pathogenesis of HCC, mediating the succinylation of serine beta-lactamase-like protein (LACTB) at K284. This finding offers a promising target for developing therapeutic strategies against HCC [69]. Similarly, other protein post-translational modifications, such as acetylation [70], ubiquitination [71], and palmitoylation [72], have been proven to be closely associated with the onset and progression of liver cancer.

Overall, our study employed TMT-labeled quantitative proteomics strategies to enhance the accuracy and reliability of protein quantification, providing an in-depth analysis of the proteomic dynamics associated with three clinical drugs used in liver cancer treatment. The research not only focused on changes in protein expression but also explored alterations in phosphorylation modifications, offering additional molecular-level insights. Through bioinformatics analysis, we gained a deeper understanding of the molecular mechanisms underlying drug action and proposed potential drug combination strategies, offering new directions for future treatment. By integrating clinical phosphorylation site data with experimental results, the study’s clinical relevance was enhanced. Despite these advances, there are limitations in our research. Our methodology predominantly relies on expression profile data to extract information regarding phosphorylation modifications. This reliance may lead to limited identification depth and insufficient exploration of the comprehensiveness and intricacy of modifications. Moreover, the pathogenesis of liver cancer is associated with diverse PTMs, which affect the proliferation and migration capabilities of liver cancer cells via multiple signaling pathways, facilitating disease initiation and progression [73]. Our study mainly focuses on phosphorylation, with insufficient investigation into other types of PTMs, potentially underestimating their important roles in liver cancer progression. Although this study proposes potential drug combination strategies, the complexity of drug interactions requires further in vivo and in vitro experiments for validation. We plan to conduct a series of experiments to verify the key findings of our proteomics data, with a deeper enrichment of phosphorylation and other PTMs. These efforts will be integrated with protein expression data to explore potential mechanisms of sensitivity and resistance, as well as additional potential drug combinations. These initiatives will be pursued in future research.

## 5. Conclusions

Our study revealed the common and specific metabolic pathways, biological processes, and protein interaction regulatory networks influenced by the clinical targeted drugs apatinib, regorafenib, and lenvatinib at the cellular level and identified potential synergistic drug combinations. Additionally, we identified several phosphorylation sites that may be closely related to the development and progression of liver cancer. In summary, our research elucidated the protein molecular changes and phosphorylation post-translational modifications induced by these clinically administered liver cancer drugs, providing valuable insights and strategies for precision medicine in HCC.

## Data Availability

The mass spectrometry proteomics data have been deposited into the ProteomeXchange Consortium (https://proteomecentral.proteomexchange.org/, accessed on 23 October 2024) via the iProX partner repository [74,75] with the dataset identifier PXD057086.

## Supplementary Materials

The following supporting information can be downloaded at: https://www.mdpi.com/article/10.3390/biomedicines13010152/s1, Figure S1: The protein–protein interaction network under the treatment of apatinib and lenvatinib. The protein–protein interaction network of upregulated and downregulated proteins in (a) apatinib-treated and (b) lenvatinib-treated groups; Figure S2: Overview of functional phosphorylation site RRM2: S20. (a) Visualization of phosphorylation site in the RRM2: S20 protein structure model. (b) Box plot illustrating differential expression of RRM2: S20 in tumors versus NATs. (c) Survival probability analysis of phosphorylation site RRM2: S20 associated with poor prognosis; Table S1: List of upregulated and downregulated proteins in the HepG2 cell line treated with apatinib, regorafenib, and lenvatinib; Table S2: List of upregulated and downregulated phosphorylation sites in the HepG2 cell line treated with apatinib, regorafenib, and lenvatinib.
