# Supplementary material for: Proteomic Characterization of Liver Cancer Cells Treated with Clinical Targeted Drugs for Hepatocellular Carcinoma
Source: Biomedicines. 2025 Jan 9;13(1):152. doi: 10.3390/biomedicines13010152 (PMC11760458; doi:10.3390/biomedicines13010152)
Supplement: Supplementary file 1 [file biomedicines-13-00152-s001.zip › 8biomedicines-3340917-supplementary.pdf]

Supporting Information

Proteomic characterization of liver cancer cells treated with clinical targeted drugs for hepatocellular carcinoma

Hezhou Long [1,2,†], Jiafu Zhou [1,2,†], Changxia Zhou [3], Shuyu Xie [3,4], Jingling Wang [2], Minjia Tan [1,2,3,4,*] and Junyu Xu [1,2,3,4,*]

1 School of Pharmaceutical Sciences, Southern Medical University, Guangzhou 510515, China; longhezhou534@zidd.ac.cn (H.L.); zhoujiafu1003@zidd.ac.cn (J.Z.)

2 Zhongshan Institute for Drug Discovery, Shanghai Institute of Materia Medica, Chinese Academy of Sciences, Zhongshan 528400, China; wangjingling317@zidd.ac.cn

3 School of Chinese Materia Medica, Nanjing University of Chinese Medicine, Nanjing 210023, China; zhouchangxia1023@zidd.ac.cn (C.Z.); xieshuyu1998@163.com (S.X.)

4 State Key Laboratory of Drug Research, Shanghai Institute of Materia Medica, Chinese Academy of Sciences, Shanghai 201203, China

* Correspondence: mjtan@simm.ac.cn (M.T.); jyxu@simm.ac.cn (J.-Y.X.)

† These authors contributed equally to this work.

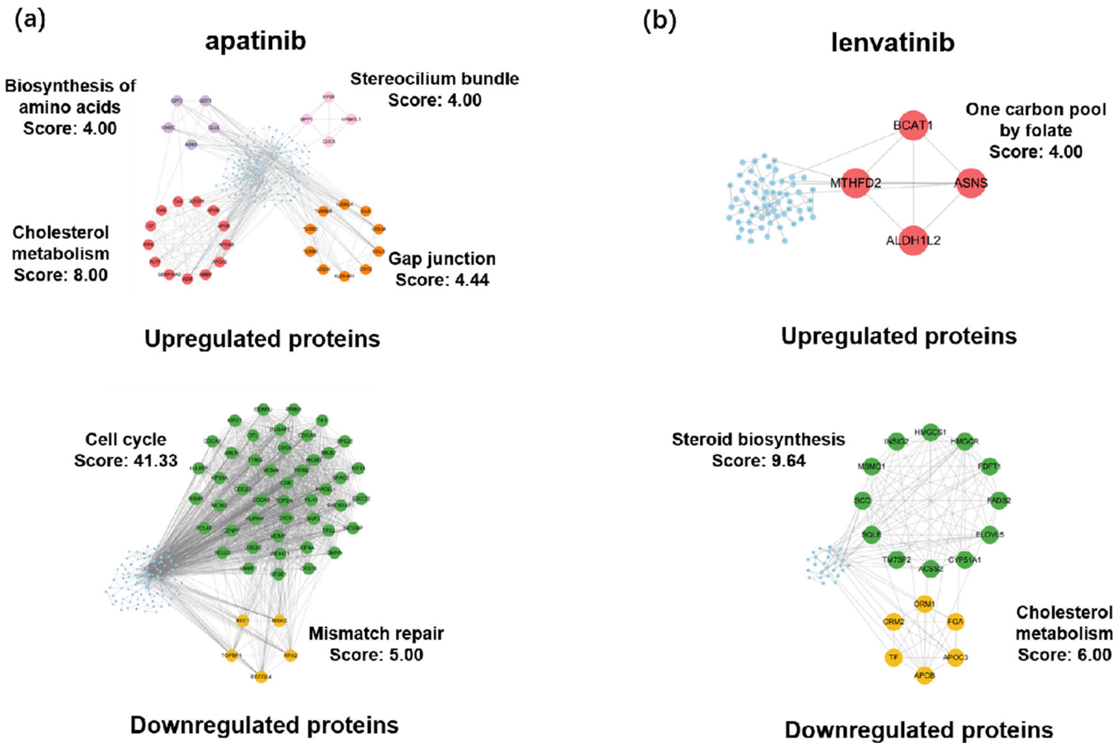

Figure S1. The protein–protein interaction network under the treatment of apatinib and lenvatinib. The protein–protein interaction network of upregulated and downregulated proteins in (**a**) apatinib-treated and (**b**) lenvatinib-treated groups.

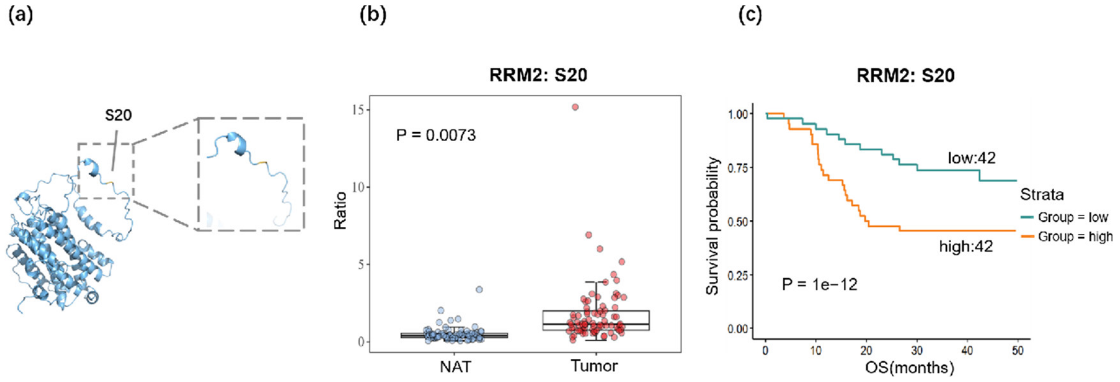

Figure S2. Overview of functional phosphorylation site RRM2: S20. (**a**) Visualization of phosphorylation site in the RRM2: S20 protein structure model. (**b**) Box plot illustrating differential expression of RRM2: S20 in tumors versus NATs. (**c**) Survival probability analysis of phosphorylation site RRM2: S20 associated with poor prognosis.

Supplementary table

Table S1. List of upregulated and downregulated proteins in the HepG2 cell line treated with apatinib, regorafenib, and lenvatinib.

Table S2. List of upregulated and downregulated phosphorylation sites in the HepG2 cell line treated with apatinib, regorafenib, and lenvatinib.
